# Comparison of open and robotic-assisted partial nephrectomy approaches using multicentric data (UroCCR-47 study)

**DOI:** 10.1038/s41598-022-22912-8

**Published:** 2022-11-08

**Authors:** A. Ingels, K. Bensalah, J. B. Beauval, P. Paparel, M. Rouprêt, H. Lang, F. X. Nouhaud, F. Hénon, F. Bruyère, F. Audenet, C. Lebacle, H. Baumert, J. A. Long, R. Tambwe, T. Charles, E. Xylinas, T. Waeckel, C. Michiels, J. Asselineau, A. Bénard, G. Margue, R. Boissier, P. Bigot, J. C. Bernhard, the Comité Cancer de l’Association Francaise d’Urologie (CCAFU), P. Gimel, Z. Khene, I. Ouzaid, N. Doumerc, C. Pettenati, F. Cornelis, P. Barthelemy, N. Rioux-Leclercq, L. Albiges, P. Bigot

**Affiliations:** 1 Department of Urology, University Hospital Henri Mondor, APHP, Créteil, France; 2 INSERM Clinical Investigation Center 1430, Henri Mondor University Hospital, AP-HP, Créteil, France; 3 Department of Urology, Rennes University Hospital, Rennes, France; 4 Department of Urology, University Hospital Rangueil, Toulouse, France; 5 Department of Urology, Lyon Sud Hospital, HCL, Pierre-Bénite, France; 6 Department of Urology, Pitié-Salpêtrière Hospital, Sorbonne University, APHP, Paris, France; 7 Department of Urology, University Hospital of Strasbourg, Strasbourg, France; 8 Department of Urology, Rouen University Hospital, Rouen, France; 9 Department of Urology, Lille University Hospital, Lille, France; 10 Department of Urology, Tours University Hospital, Tours, France; 11 Department of Urology, Hôpital Européen Georges Pompidou, APHP, Paris, France; 12 Department of Urology, Bicêtre University Hospital, APHP, Le Kremlin Bicêtre, France; 13 Department of Urology, Paris Saint-Joseph Hospital, Paris, France; 14 Department of Urology, Grenoble University Hospital, Grenoble, France; 15 Department of Urology, Reims University Hospital, Reims, France; 16 Department of Urology, Poitiers University Hospital, Poitiers, France; 17 Department of Urology, Bichat-Claude Bernard Hospital, APHP, Paris, France; 18 Department of Urology, Caen University Hospital, Caen, France; 19 Department of Urology, Hôpital Pellegrin, Bordeaux University Hospital, Bordeaux, France; 20 Clinical Epidemiology Unit (USMR), CHU Bordeaux, 33000 Bordeaux, France; 21 Department of Urology and Renal Transplantation, La Conception University Hospital, APHM, Marseille, France; 22 Department of Urology, Angers University Hospital, Angers, France; 23 French Research Network on Kidney Cancer UroCCR, Bordeaux, France; 24 CHU Henri MONDOR, 51 Avenue du Maréchal de Lattre de Tassigny, 94010 Créteil Cédex, France; 25 Department of Urology, Médipôle Saint-Roch, Avenue Ambroise Croizat, 66330 Cabestany, France; 26 Department of Urology, Hôpital Foch, 40 Rue Worth, 92150 Suresnes, France; 27 Department of Radiology, Hôpital Tenon, 4 Rue de La Chine, 75020 Paris, France; 28 Department of Oncology, ICANS, 17 Rue Albert Calmette, 67200 Strasbourg, France; 29 Department of Pathology, Rennes University Hospital, Rennes, France; 30 Department of Oncology, Institut Gustave Roussy, 114 Rue Edouard Vaillant, 94805 Villejuif, France; 31 Department of Urology, CHU Angers, All. de la Maine, 49100 Angers, France

**Keywords:** Cancer therapy, Renal cancer

## Abstract

We compared the outcomes of robotic-assisted partial nephrectomy (RPN) and open partial nephrectomy (OPN) using contemporary data to respond to unmet clinical needs. Data from patients included in the registry who underwent partial nephrectomy between January 01, 2014 and June 30, 2017 within 20 centres of the French Network for Research on Kidney Cancer UroCCR were collected (NCT03293563). Statistical methods included adjusted multivariable analyses. Rates of peri- and post-operative transfusion, and of surgical revision, were lower in the RPN (n = 1434) than the OPN (n = 571) group (2.9% vs. 6.0%, *p* = 0.0012; 3.8% vs. 11.5%, *p* < 0.0001; 2.4% vs. 6.7%, *p* < 0.0001, respectively). In multivariable analyses, RPN was independently associated with fewer early post-operative complications than OPN (overall: odds-ratio [95% confidence interval, CI] = 0.48 [0.35–0.66]; severe: 0.29 [0.16–0.54], *p* < 0.0001 for both) and shorter hospital stays (34% [30%; 37%], *p* < 0.0001). RPN was also a significantly associated with a decresedrisk of post-operative acute renal failure, and new-onset chronic kidney disease at 3 and 12 months post-surgery. There were no between-group differences in oncological outcomes. In comparison with OPN, RPN was associated with improved peri- and post-operative morbidity, better functional outcomes, and shorter hospital stays. Our results support the use of RPN, even for large and complex tumours.

## Introduction

According to the guidelines of the French and European associations of urology, partial nephrectomy (PN) is the standard of care for localised kidney T1a (≤ 4 cm) tumours^1,2^. When technically possible, PN is also the standard of care in case of imperative surgery (solitary kidney, bilateral disease, and pre-existing renal insufficiency)^1^, and may be preferred in case of localised T1b and T2 tumours, depending on their complexity^1,2^. Recent data have shown that PN might also represent a feasible option for large kidney tumours (> 7 cm), with substantial but acceptable morbidity, limited risk of local recurrence, and excellent preservation of renal function^3^.

The open approach has long been considered the gold standard for PN. Although the laparoscopic route was popularized in the 2000s, technical difficulties (primarily longer arterial clamping times with ischaemia) limited its development^4^. However, the introduction of robotic assistance bypassed these limitations and led to an increasing proportion of renal cell carcinoma surgery being performed using minimally invasive procedures. In France, Ouzaid et al.^5^ reported that the annual number of robotic-assisted partial nephrectomy (RPN) procedures continuously increased between 2009 and 2015, whereas the number of laparoscopic partial nephrectomy (LPN) procedures remained stable over this period. Several systematic reviews and meta-analyses have highlighted the benefits of RPN compared to open partial nephrectomy (OPN)^6–11^ and LPN^12–14^. According to Autorino and Porpiglia^15^, RPN is becoming “*the new gold standard for nephron sparing surgery*” (NSS). However, the French health care system does not have a specific pricing system for this approach, and therefore does not reimburse the additional costs involved.

Data from the literature are usually based on retrospective series involving single centres and thus few of these data are contemporary and truly representative of French practices. The aim of our study was to use contemporary data, prospectively collected within the French Network for Research on Kidney Cancer UroCCR, to compare short- and mid-term PN outcomes according to the surgical approach. The recent report of the French National Authority for Health (HAS) on robotic-assisted nephrectomy indicated that RPN should be compared to OPN rather than LPN, due to the technical difficulties associated with the LPN approach^16^. Thus, in this article we report on the comparison between OPN and RPN outcomes.

## Methods

### Study design and setting

CONTEMPORARI-PN (Comparative analysis of cONtemporary partial nEphrectoMy outcoMes between oPen, laparascOpic and Robotic AppRoaches In France) was a French, retrospective, observational, multicentre, cohort study based on the analysis of prospectively collected data from the UroCCR database (NCT03293563; CNIL DR 2013-206;). Informed consent was obtained from all participants. Collected data included patient and tumour characteristics, as well as surgery-related information. Partial nephrectomy could have been performed through OPN, LPN, or RPN. Thus, two-by-two comparisons were performed using a statistical model integrating these three arms: OPN, LPN, and RPN. However, only the comparisons between RPN and OPN are presented here.

### Participants and procedures

All patients iwho underwent PN for kidney cancer and consented to be included in the database between January 01, 2014 and June 30, 2017 within 20 centres belonging to the UroCCR network were included into analyses. The surgical approach was chosen at the surgeon’s discretion. All procedures were performed by experienced surgical teams. RPN was performed using the da Vinci^®^ Surgical System (Intuitive Surgical Sunnyvale, CA, USA).

### Outcomes and assessment methods

#### Primary outcome

The peri-operative morbidity of OPN and RPN was evaluated by measuring the rates of early post-operative surgical complications (overall and severe according to the Clavien-Dindo scale^17^) and late post-operative complications occurring within 30 days.

#### Secondary outcomes

The peri-operative morbidity of OPN and RPN was also evaluated by measuring the rates of peri-operative and post-operative transfusion, surgical re-operation, and post-operative death within 30 days.

Oncological outcomes were evaluated by assessing the rates of positive surgical margins, local and contralateral recurrence, metastatic progression, recurrence-free survival, and overall survival.

Functional outcomes were evaluated before surgery and post-operatively on days 1–3 (D1–D3), and at month 3 (M3) and M12 by measuring the following parameters: creatinine, glomerular filtration rate (GFR) calculated according to the modified diet and renal disease (MDRD) formula, new-onset chronic kidney disease (CKD) for patients with pre-operative stage I or II CKD defined by a eGFR < 60 mL/min/1.73 m^2^, and post-operative acute renal failure rates. We considered an acute renal failure when the eGFR shifted under 60 mL/min/1.73 m^2^. We also analysed the creatinaemia relative change ((creatinaemia after surgery–creatinaemia before surgery)/creatinaemia before surgery) and GFR relative change ((GFR after surgery–GFR before surgery)/GFR before surgery) that could be more clinically significant than new-onset chronic kidney disease.

Length of hospital stay, and Trifecta achievement according to the Khalifeh criteria^18^ (defined as negative surgical margins + zero peri-operative complications and a warm ischaemia time ≤ 25 min) were also assessed.

### Statistical analyses

Statistical analyses were carried out using SAS software version 9.3 (SAS Institute Inc., NC, USA). Qualitative variables were expressed as numbers and percentages. Quantitative variables were expressed as means and standard deviations. Early and late post-operative surgical complications, qualitative functional outcomes and Trifecta were compared between groups using mixed-effects logistic regression models, with the study centre as a random effect. Local and contralateral recurrence, and metastatic progression were described using the cumulative incidences approach to take into account the competing risk of death occurring before observing these events. Recurrence-free and overall survival were described using the Kaplan–Meier method. Oncological outcomes were compared between groups using stratified (according to study centre) Cox proportional-hazards models. Quantitative functional outcomes and length of hospital stay were compared between groups using mixed linear regression models, with the study centre as a random effect. Model assumptions were systematically verified. Quantitative functional outcomes and length of hospital stay were transformed into natural logarithms to comply with model assumptions. Removal of the random effect (for logistic and linear regression models) and/or use of the Firth’s penalization method (for Cox and logistic models) was proposed when models did not converge. All comparisons were done with and without adjustment for the following prognostic factors: tumour size, imperative NSS indication, age at surgery, American Society of Anesthesiologists (ASA) score, body mass index (BMI) at diagnosis and pre-operative creatininemia for post-operative acute renal failure and new onset CKD only. The RENAL score^19^ was not included in the model because variations in tumour size had already been taken into account as a prognostic factor. Stratified analyses were performed according to tumour size (≤ 4 cm, [4–7] cm, > 7 cm) and NSS indication. Rates of peri-operative transfusion, surgical re-operation and post-operative deaths within 30 days were compared between groups using the Chi-squared or Fisher exact tests, depending on the event distribution. The type I error rate was set at 5% for all comparisons. No missing data management strategy was used.

### Ethics approval and consent to participate

This study was performed in line with the principles of the 1964 Helsinki Declaration and its later amendments or comparable ethical standards. In accordance with French law, the processing of personal data was approved by the French data protection agency (*Commission Nationale de l'Informatique et des Libertés, CNIL*): authorization DR 2013-206 and DR 2014-251. Moreover, the UroCCR project and this particular study (NCT03293563) were IRB-approved (*Comité de Protection des Personnes (CPP) Sud-Ouest et Outre mer III*, decision DC 2012/108). All patients gave their written consent before inclusion in the UroCCR database, after having received oral and written information about the UroCCR project.

## Results

Of the 2097 patients included int the study, who underwent PN for cT1-2N0M0 tumours between January 01, 2014 and June 30, 2017, 1969 (93.9%) underwent either OPN (N = 560) or RPN (N = 1409). Patient and tumour characteristics, as well as surgical outcomes are presented in Table 1.

**Table 1:** Patient and tumour characteristics, surgical, functional and oncologic outcomes according to the surgical approach (*N* = 1969).

	OPN (N = 560)	RPN (N = 1409)
Age^a^ (years), mean ± SD (*m* = 4)	59.2 ± 13.3	59.9 ± 12.4
**Gender**^**a**^**, n/N (%)**		
Male	359/560 (64.1)	951/1409 (67.5)
Female	201/560 (35.9)	458/1409 (32.5)
BMI^a^, mean ± SD (*m* = 67)	26.8 ± 5.2	27.0 ± 5.4
**ASA score**^**a**^**, n/N (%) (*m* = 170)**		
1	168/496 (33.9)	339/1303 (26.0)
2	261/496 (52.6)	768/1303 (58.9)
3	61/496 (12.3)	193/1303 (14.8)
4	6/496 (1.2)	3/1303 (0.2)
Baseline kidney function mean ± SD (*m* = 390)	87.5 (30.5)	83.9 (27.0)
Solitary kidney^a^, n/N (%) (*m* = 19)	41/548 (7.5)	55/1402 (3.9)
Tumour size^a^ (cm), mean ± SD (*m* = 21)	4.4 ± 2.5	3.4 ± 1.6
**Tumour size**^**a**^**, n/N (%) (*m* = 21)**		
≤ 4 cm	342/620 (55.2)	1074/1484 (72.4)
[4–7] cm	205/620 (33.1)	367/1484 (24.7)
> 7 cm	73/620 (11.8)	43/1484 (2.9)
**RENAL score**^**a,b**^**, n/N (%) (*m* = 244)**		
Low complexity	172/565 (30.4)	576/1316 (43.8)
Moderate complexity	249/565 (44.1)	582/1316 (44.2)
High complexity	144/565 (25.5)	158/1316 (12.0)
**NSS indication**^**a**^**, n/N (%) (*m* = 159)**		
Elective	310/499 (62.1)	1026/1347 (76.2)
Imperative	138/499 (27.7)	204/1347 (15.1)
Relative	51/499 (10.2)	117/1347 (8.7)
Follow-up duration^a,c^ (months), mean ± SD	8.9 ± 5.1	8.6 ± 5.3
**Number of surgeries/patient**^**a,d**^**, n/N (%)**		
1	544/560 (97.1)	1390/1409 (98.7)
2 (same surgery)	8/560 (1.4)	17/1409 (1.2)
2 (other surgery)^c^	8/560 (1.4)	2/1409 (0.1)
Surgery duration (min), mean ± SD	129.6 ± 70.0	144.5 ± 74.1
Clamping, n/N (%) (*m* = 40)	500/555 (90.1)	1295/1410 (91.8)
Clamp time (min), mean ± SD (*m* = 35)	17.6 ± 7.5	17.4 ± 8.9
Blood loss (mL), mean ± SD (*m* = 66)	371.2 ± 458.9	262.5 ± 343.3
Length of hospital stay (days), mean ± SD (*m* = 172)	7.1 ± 4.2	4.2 ± 3.3
Acute renal failure	26/486 (5.3)	9/1393 (0.6)
12 months chronic kidney disease onset n/N (%)	16/65 (24.6)	22/224 (9.8)
Local and loco-regional recurrence n/N (%)	16/365 (4.4)	22/1195 (1.8)
Metastatic progression n/N (%)	16/363 (4.4)	29/1191 (2.4)

ASA American Society of Anesthesiologists, *BMI* body mass index, *m* missing data, *NSS* nephron sparing surgery, *OPN* open partial nephrectomy, *RPN* robotic-assisted partial nephrectomy, *SD* standard deviation.

^a^Patient characteristics at first surgery.

^b^Defined by Kutikov et al.^18^.

^c^For patients with data from at least one follow-up visit: OPN, N = 390; RPN, N = 1263.

^d^Data for one additional patient, who underwent OPN as a second surgical approach after a laparoscopic partial nephrectomy, were excluded from most analyses. However, their data were included in the descriptive statistics for surgery (this table), and in the comparative analyses in which surgery was the statistical unit (Table 2—except oncological outcomes and Table 3).

### Peri-operative and post-operative morbidity

Rates of early post-operative complications were lower in patients who underwent RPN than in those that underwent OPN (overall: 17.9% vs. 34.9%; severe: 2.0% vs. 5.5%), whereas no between-group differences were observed for late post-operative complications (4.6% vs. 6.5%). Multivariable analyses showed that RPN was independently associated with a decrease in both overall (odds-ratio [95% confidence interval], OR 95% CI = 0.48 [0.35–0.66], *p* < 0.0001) and severe (OR 95% CI = 0.29 [0.16–0.54], *p* < 0.0001) early post-operative complications compared with OPN (Table 2). Stratified analyses did not show any impact of tumour size or NSS indication on this primary outcome (Supplementary Table 1). Rates of peri- and post-operative transfusion, and of re-operation were lower in patients who underwent RPN than in those that underwent OPN (Table 3).

**Table 2:** Post-operative complications, oncological outcomes and functional outcomes in patients undergoing RPN versus OPN—univariable and multivariable analyses.

	Univariable analysis	N	*p* value	Multivariable analysis^a^	N	*p* value
**Post-operative complications, OR [95% CI]**						
Early, all grades	0.41 [0.32–0.52]	1371/444	< .0001	0.48 [0.35–0.66]	1173/333	< 0.0001
Early, severe^b,c^	0.35 [0.20–0.61]	1360/439	0.0002	0.29 [0.16–0.54]	1158/333	< 0.0001
Late, all grades^c,d^	0.70 [0.43–1.14]	1274/387	0.1523	0.75 [0.43–1.30]	1108/309	0.3044
**Oncological outcomes**						
Positive surgical margins, OR [95% CI]	0.84 [0.53–1.35]	1291/452	0.4690	0.97 [0.51–1.85]	1127/340	0.9321
Local recurrence rate, HR [95% CI]	0.43 [0.22–0.81]	1195/365	0.0094	0.48 [0.20–1.15]	1046/291	0.0976
Contralateral recurrence rate^e^, HR [95% CI]	0.39 [0.10–1.51]	1195/365	0.1751	0.52 [0.08–3.37]	1046/291	0.4909
Metastatic progression rate, HR [95% CI]	0.65 [0.35–1.24]	1191/361	0.1934	0.76 [0.32–1.82]	1043/291	0.5392
Recurrence-free survival rate, HR [95% CI]	0.53 [0.34–0.84]	1192/364	0.0063	0.56 [0.30–1.03]	1044/292	0.0606
Overall survival rate, HR [95% CI]	0.45 [0.14–1.42]	1261/388	0.1744	0.29 [0.06–1.44]	1100/309	0.1292
**Functional outcomes**						
Creatininaemia relative changes (%), estimation [95% CI]^f^						
Before surgery/D1–D3	− 2.28 [− 3.2;− 1.35]	1111/328	< 0.0001	− 1.93 [− 2.98;− 0.88]	982/263	0.0003
Before surgery/M3^g^	− 0.64 [− 3.45;2.17]	253/38	0.6547	− 0.47 [− 3.51;2.57]	243/36	0.7599
Before surgery/M12^g^	0.04 [− 1.88;1.95]	248/82	0.9682	0.19 [− 1.93;2.31]	230/72	0.8627
GFR relative changes(%), estimation [95% CI]^f^						
Before surgery/D1–D3	0.40 [− 0.49;1.28]	1063/323	0.3794	0.35 [− 0.69;1.38]	948/260	0.5098
Before surgery/M3^g^	1.46 [− 0.47;3.40]	266/39	0.1372	1.14 [− 0.93;3.20]	253/37	0.2805
Before surgery/M12	0.33 [− 1.07;1.73]	259/82	0.6437	0.11 [− 1.40;1.62]	241/72	0.8837
Post-operative acute renal failure^c^, OR [95% CI]	0.12 [0.06–0.25]	1393/486	< 0.0001	0.31 [0.13–0.77]	974/272	0.0111
New-onset CKD^h^, OR [95% CI]						
D1–D3	0.71 [0.49–1.02]	935/260	0.0634	0.88 [0.50–1.55]	830/203	0.6623
M3^c^	0.14 [0.06–0.35]	228/30	<0.0001	0.30 [0.11–0.85]	218/28	0.0233
M12^c^	0.33 [0.16–0.68]	224/65	0.0025	0.37 [0.15–0.91]	207/58	0.0304
Trifecta^i^, OR [95% CI]	1.21 [0.94–1.55]	1275/442	0.1339	0.73 [0.51–1.05]	1116/336	0.0879
Length of hospital stay, estimation [95% CI]^j^	0.62 [0.59–0.65]	1380/453	< 0.0001	0.66 [0.63–0.70]	1183/337	< 0.0001

*N* frequency (RPN/OPN), *CI* confidence interval, *CKD* chronic kidney disease, *D* day, *GFR* glomerular filtration rate, *HR* hazard ratio, *M* month, *OR* odds ratio.

^a^Adjusted for the following prognostic factors: tumour size, imperative NSS indication, age at surgery, ASA score, BMI at diagnosis, and pre-operative creatininemia for post-operative acute renal failure and new-onset CKD only.

^b^Surgical complications only. When the Clavien-Dindo grade^17^ was not provided, data were considered to be missing.

^c^As the mixed effects regression model failed to converge, the Firth’s penalization method was used.

^d^Occurrence of at least one complication within 30 days post-surgery.

^e^As the survival model failed to converge, the Firth’s penalization method was used.

^f^To comply with model hypotheses, relative changes were calculated after transformation of the measured values into natural logarithms.

^g^As the mixed effects regression model failed to converge, the random effect on the study centre was removed.

^h^For patients with pre-operative stage I or II CKD only.

^i^Trifecta was defined as negative surgical margins + zero peri-operative complications and a warm ischaemia time (WIT) ≤ 25 min.

^j^To comply with model hypotheses, data for the length of hospital stay were transformed into natural logarithms. Estimations were the exponential of the model parameter, interpreted as a multiplicative factor: < 1 (> 1) means a shorter (longer) length of stay with RPN compared to OPN.

**Table 3:** Peri-operative morbidity secondary outcomes according to the surgical approach (*N* = 2005 surgeries).

	OPN (N = 571)	RPN (N = 1434)	*p* value
Peri-operative transfusion, n/N (%) (*m* = 54)	33/547 (6.0)	41/1404 (2.9)	0.0012^a^
Post-operative transfusion, n/N (%) (*m* = 173)	52/454 (11.5)	52/1378 (3.8)	< 0.0001^a^
Surgical re-operation rate, n/N (%) (*m* = 149)	31/462 (6.7)	34/1394 (2.4)	< 0.0001^a^
Post-operative death within 30 days, n/N (%) (*m* = 325)	2/397 (0.5)	2/1283 (0.2)	0.2387^b^

*m* missing data, *OPN* open partial nephrectomy, *RPN* robotic-assisted partial nephrectomy.

^a^Chi-squared test.

^b^Fisher exact test.

### Oncological outcomes

Few patients had positive surgical margins (OPN, N = 29/452, 5.8%; RPN, N = 63/1291, 4.9%), and no significant difference was observed between groups (Table 2). Cumulative incidence curves and Kaplan–Meier curves of oncologic outcomes are shown in Supplementary Fig. 1. Multivariable analyses showed no between-group differences (Table 2).

### Functional outcomes

Analyses of functional outcomes are presented in Table 2. Pre-operative/D1-D3 changes in creatininaemia were significantly smaller for the RPN group than for the OPN group. No between-group differences in estimated GFR changes were observed at any time post-surgery. In comparison with OPN, RPN was significantly associated with a lower risk of post-operative acute renal failure, and from new-onset CKD at M3 and M12 post-surgery. Compared with OPN, multivariable analysis revealed a trend towards better fulfilment of Trifecta outcomes with RPN (OR [95% CI] = 0.73 [0.51–1.05], *p* = *0.0879*), and stratified analyses showed that Trifecta outcomes were more likely to be fulfilled with RPN for tumours > 7 cm (Supplementary Table 1).

Finally, RPN was associated with a 34% reduction in the length of hospital stay compared to OPN (Table 2).

## Discussion

This analysis of prospectively collected data from almost 2,000 patients who underwent PN over 3.5 years in 20 centres, showed a clear benefit of RPN over OPN in terms of early post-operative complications, peri- and post-operative transfusion rates, re-operation rates, length of hospital stay, pre-operative/D1-D3 creatininaemia changes, post-operative acute renal failure, and new-onset CKD at M3 and M12 post-surgery. No between-group differences were observed for late complication rates, positive surgical margins, and GFR changes. The surgical approach did not have any impact on oncological outcomes.

Our study responds to unmet clinical needs, and addresses the concerns of the HAS surrounding the lack of prospective contemporary studies on RPN^16^. Indeed, previous prospective studies included few patients and/or did not include patients consecutively, with RPN groups being added a posteriori^16^. Thus, in such studies, including a previous French study^20^ in which patients were included over an extensive time period (2006–2014), the observed differences between RPN and OPN groups may not have been uniquely related to the surgery: there may have been differences at several levels of the patient care pathway including anaesthesia, management in the intensive care unit, and other post-operative parameters. Additionally, this previous French study^20^ included RPN performed by surgeons with various levels of expertise, with most of them still being on the RPN learning curve. This was not the case in our study.

Our contemporary results are in line with those reported by several systematic reviews and/or meta-analyses^6,9–11,21^, except for estimated GFR outcomes, which were reported as better in patients who underwent RPN in two systematic reviews and meta-analyses^9,11^. Creatininaemia changes, post-operative acute renal failure, and new-onset CKD could not be compared with the literature due to a lack of published data.

Multivariable analyses showed that the benefit of RPN over OPN regarding early post-operative complications (overall or severe), post-operative acute renal failure, and new-onset CKD at M3 and M12 post-surgery was independent of the tumour size, imperative NSS indication, age at surgery, ASA score, and BMI at diagnosis. Interestingly, the superiority of RPN over OPN on Trifecta (which included zero peri-operative complications) was observed for tumours > 7 cm in stratified analyses. RPN therefore had a beneficial impact on these parameters, even for large and complex tumours. The seemingly greater benefit of RPN observed for new-onset CKD at M3 post-surgery for tumours > 7 cm may be related to the higher probability of occurrence of this event in patients with large tumours. The benefits of RPN over OPN for complex tumours have also been highlighted in a systematic review (RENAL score ≥ 7)^11^ and in a single centre study (RENAL score > 9)^22^, with reported benefits including reductions in blood loss and length of stay, as well as in intra-operative complications and transfusion rates^22^, and post-operative complications^11,22^. In contrast, apart from a shorter hospital stay after RPN versus OPN, Zargar et al. did not identify any between-group differences in their preliminary study focusing on solitary kidney^23^. To the best of our knowledge, the benefits of RPN for achieving Trifecta for large and complex tumours have not been demonstrated previously.

Several authors concluded their systematic review and meta-analysis on PN by claiming that randomized controlled trials (RCTs) to compare RPN and OPN would be needed to confirm their findings^6,9,10,21^. However, the implementation of such RCTs is no longer conceivable due to the rapid expansion of RPN, and ethical issues. In France, some centres already only use the robotic approach for PN, convinced of the net benefits of this surgical approach, both for patients and hospitals. Outpatient RPN has even been implemented in Bordeaux for selected patients, supported by a dedicated, nurse-led clinical pathway, and has been shown to provide a high level of patient satisfaction as well as economic optimization of robotic assistance^24,25^.

Our study had some limitations. Two-by-two comparisons were performed using a statistical model integrating an initial three study arms: OPN, LPN, and RPN. However, the results of the multivariable and stratified analyses remained stable after removal of the LPN arm, enabling us to report only on the clinically relevant comparison between OPN and RPN. The retrospective design of the study might lead to selection biases with more complex tumors and more imperative indications in the OPN arm. Although these differences were tackled with the multivariate analyses, other factors and unmeasured cofounders might affect the results and selection bias remains a concern. The exclusive practice of RPN in some of our study centres may have introduced a bias because no same-centre comparisons of outcomes were possible. However, our study was multicentric and gathered data from 20 centres, all of which were expert centres for both RPN and OPN. Furthermore, the impact of surgeons’ expertise on outcomes also needs to be considered. Although this is a factor that cannot be eliminated, our study, like most previous evaluations of RPN, used data generated by high-volume surgeons with extensive experience. Our findings may not therefore be readily transferable to settings were RPN is conducted by less experienced surgeons. Another question to be addressed is the comparative cost-effectiveness of the two surgical approaches. Our findings demonstrated that patients who underwent RPN had shorter hospital stays, fewer complications and re-operations than those who underwent OPN. Other previously reported advantages of minimally invasive PN over OPN that may also have some impact on costs include lower rates of peri-operative opioid use and fewer days of workplace absenteeism^26^. These advantages could balance out the cost of robotic assistance. In their meta-analysis, Wu et al.^6^ found that the overall cost of RPN was not significantly higher than that of OPN, and Bernhard et al.^25^ recently reported that implementing RPN along with enhanced recovery after surgery and day-case nurse-led protocols may facilitate the economic sustainability of robotic assistance for hospitals where the extra cost is not covered by the healthcare system.

In conclusion, RPN was associated with improved peri- and post-operative morbidity, shorter hospital stays, better functional outcomes for some parameters, and similar oncological outcomes in comparison with OPN in our multicentre contemporary study. Our results support the use of RPN, even for large and complex tumours.

## Supplementary Information

Supplementary Information 1.

Supplementary Information 2.

Supplementary Information 3.

## Data Availability

Data are available from the corresponding author upon request.
