# Supplementary material for: Comparison of open and robotic-assisted partial nephrectomy approaches using multicentric data (UroCCR-47 study)
Source: Sci Rep. 2022 Nov 8;12:18981. doi: 10.1038/s41598-022-22912-8 (PMC9643517; doi:10.1038/s41598-022-22912-8)
Supplement: Supplementary file 1 — Supplementary Information 1. [file 41598_2022_22912_MOESM1_ESM.pdf]

Supplementary Fig. 1 Cumulative incidence curves of local (A) and contralateral (B) recurrence, and metastatic progression (C), and Kaplan-Meier curves of recurrence-free (D) and overall (E) survival in patients subjected to open partial nephrectomy (OPN, full line) versus robotic partial nephrectomy (RPN, dashed line).

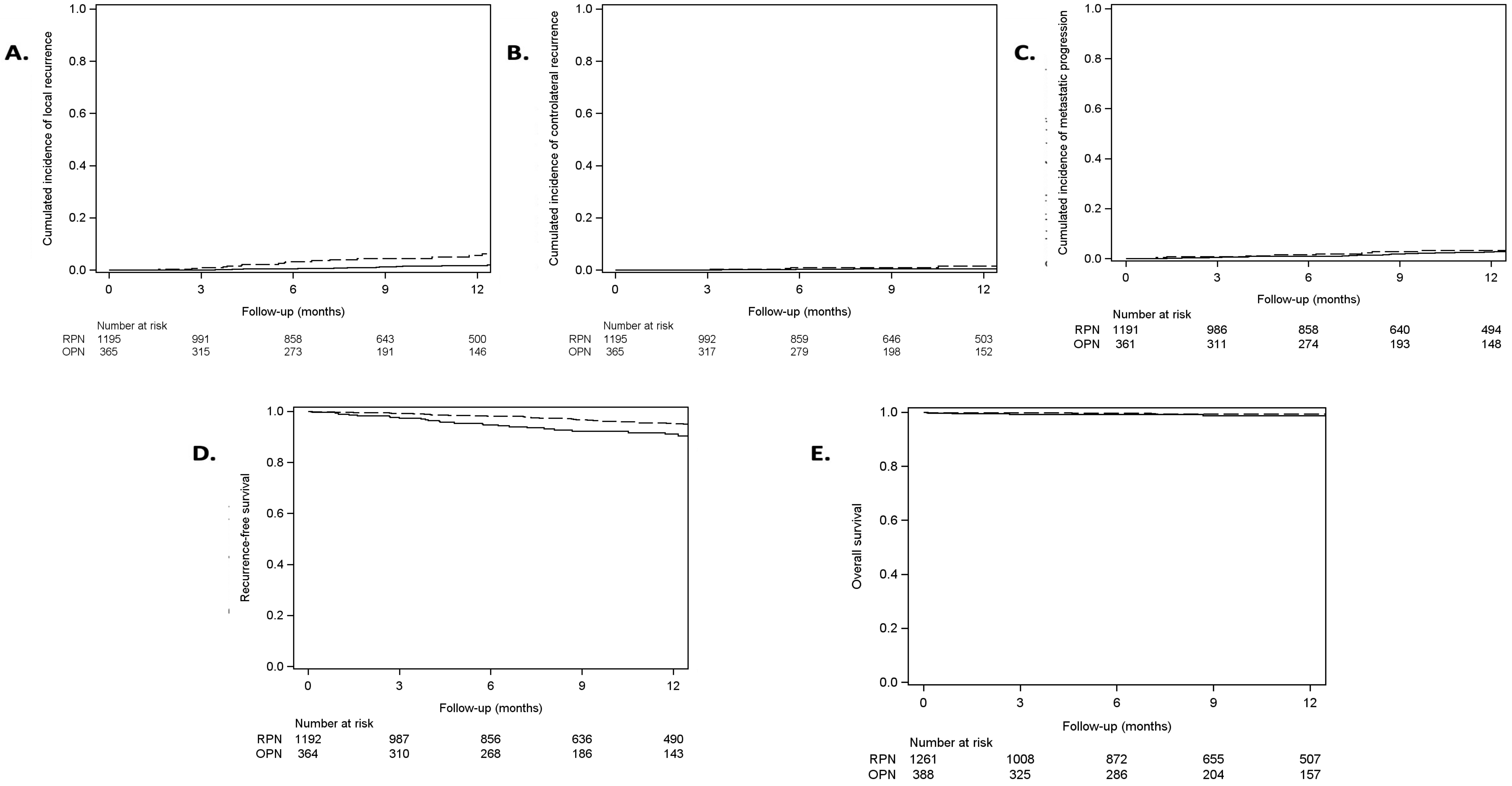
A.
Cumulated incidence of local recurrence
1.0
0.8
0.6
0.4
0.2
0.0
0 3 6 9 12
Follow-up (months)
Number at risk
RPN 1195 991 858 643 500
OPN 365 315 273 191 146
B.
Cumulated incidence of controlateral recurrence
1.0
0.8
0.6
0.4
0.2
0.0
0 3 6 9 12
Follow-up (months)
Number at risk
RPN 1195 992 859 646 503
OPN 365 317 279 198 152
C.
Cumulated incidence of metastatic progression
1.0
0.8
0.6
0.4
0.2
0.0
0 3 6 9 12
Follow-up (months)
Number at risk
RPN 1191 986 858 640 494
OPN 361 311 274 193 148
D.
Recurrence-free survival
1.0
0.8
0.6
0.4
0.2
0.0
0 3 6 9 12
Follow-up (months)
Number at risk
RPN 1192 987 856 636 490
OPN 364 310 268 186 143
E.
Overall survival
1.0
0.8
0.6
0.4
0.2
0.0
0 3 6 9 12
Follow-up (months)
Number at risk
RPN 1261 1008 872 655 507
OPN 388 325 286 204 157
