# Supplementary material for: Comparison of open and robotic-assisted partial nephrectomy approaches using multicentric data (UroCCR-47 study)
Source: Sci Rep. 2022 Nov 8;12:18981. doi: 10.1038/s41598-022-22912-8 (PMC9643517; doi:10.1038/s41598-022-22912-8)
Supplement: Supplementary file 2 — Supplementary Information 2. [file 41598_2022_22912_MOESM2_ESM.docx]

**Supplementary Table 1** Stratified analyses

|  | **Multivariable analysis** | **Interaction *p* value** |
| --- | --- | --- |
| **Post-operative complications**, OR [95% CI] |  |  |
| Early post-operative complications (overall) |  |  |
| Tumour size ≤4 cm | 0.41 [0.28-0.62] | *0.7681* |
| Tumour size ]4–7] cm | 0.58 [0.34-0.97] |  |
| Tumour size >7 cm | 0.64 [0.26-1.61] |  |
| Non-imperative NSS indication | 0.50 [0.36-0.70] | *0.7496* |
| Imperative NSS indication | 0.40 [0.24-0.67] |  |
| Early post-operative complications (severe)^b,c^ |  |  |
| Tumour size ≤4 cm | 0.30 [0.13-0.66] | *0.9802* |
| Tumour size ]4–7] cm | 0.36 [0.12-1.06] |  |
| Tumour size >7 cm | 0.21 [0.03-1.32] |  |
| Non-imperative NSS indication | 0.30 [0.15-0.6] | *0.9667* |
| Imperative NSS indication | 0.29 [0.08-1.02] |  |
| Late post-operative complications (overall)^c,d^ |  |  |
| Tumour size ≤4 cm | 0.67 [0.34-1.32] | *0.4121* |
| Tumour size ]4–7] cm | 0.65 [0.25-1.70] |  |
| Tumour size >7 cm | 7.82 [0.40-151.5] |  |
| Non-imperative NSS indication | 0.58 [0.28-1.19] | *0.6304* |
| Imperative NSS indication | 0.99 [0.40-2.46] |  |
| **Oncological outcomes** |  |  |
| Positive surgical margins^c^, OR [95% CI] |  |  |
| Tumour size ≤4 cm | 0.74 [0.34-1.61] | *0.8066* |
| Tumour size ]4–7] cm | 0.87 [0.4-1.9] |  |
| Tumour size >7 cm | 1.19 [0.12-12.17] |  |
| Non-imperative NSS indication | 0.74 [0.41-1.33 | *0.3785* |
| Imperative NSS indication | 1.33 [0.35-5.00] |  |
| Local recurrence rate^e^, HR [95% CI] |  |  |
| Tumour size ≤4 cm | 0.19 [0.06-0.57] | *0.2107* |
| Tumour size ]4–7] cm | 1.26 [0.38-5.07] |  |
| Tumour size >7 cm | 0.63 [0.05-5.34] |  |
| Non-imperative NSS indication | 0.43 [0.15-1.36] | *0.9724* |
| Imperative NSS indication | 0.52 [0.15-1.65] |  |
| Contralateral recurrence^e^, HR [95% CI] |  |  |
| Tumour size ≤4 cm | 0.29 [0.02-4.03] | *0.8821* |
| Tumour size ]4–7] cm | 1.11 [0.06-19.77] |  |
| Tumour size >7 cm | 0.38 [0.01-25.55] |  |
| Non-imperative NSS indication | 0.22 [0.02-2.96] | *0.7376* |
| Imperative NSS indication | 0.73 [0.08-6.52] |  |
| Metastatic progression rate^e^, HR [95% CI] |  |  |
| Tumour size ≤4 cm | 0.43 [0.12-1.51] | *0.7713* |
| Tumour size ]4–7] cm | 0.88 [0.21-3.59] |  |
| Tumour size >7 cm | 0.92 [0.23-3.67] |  |
| Non-imperative NSS indication | 0.83 [0.32-2.48] | *0.7860* |
| Imperative NSS indication | 0.55 [0.12-2.12] |  |
| Recurrence-free survival^e^, HR [95% CI] |  |  |
| Tumour size ≤4 cm | 0.33 [0.15-0.75] | *0.4824* |
| Tumour size ]4–7] cm | 0.80 [0.32-2.00] |  |
| Tumour size >7 cm | 0.77 [0.23-2.61] |  |
| Non-imperative NSS indication | 0.49 [0.25-1.04] | *0.8635* |
| Imperative NSS indication | 0.66 [0.27-1.60] |  |
| Overall survival^e^, HR [95% CI] |  |  |
| Tumour size ≤4 cm | 0.42 [0.04-4.61] | *0.8867* |
| Tumour size ]4–7] cm | 0.22 [0.03-1.84] |  |
| Tumour size >7 cm | 1.40 [0.01-155.10] |  |
| Non-imperative NSS indication | 0.16 [0.02-1.02] | *0.4995* |
| Imperative NSS indication | 0.83 [0.08-8.41] |  |
| **Functional outcomes** |  |  |
| Creatininaemia relative changes (Before surgery/D1-D3), estimation [95% CI] |  |  |
| Tumour size ≤4 cm | -2.51 [-3.85;-1.16] | *0.1496* |
| Tumour size ]4–7] cm | -0.90 [-2.57;0.77] |  |
| Tumour size >7 cm | -1.58 [-4.76;1.6] |  |
| Non-imperative NSS indication | -1.90 [-3.11;-0.68] | *0.6636* |
| Imperative NSS indication | -1.98 [-3.87;-0.1] |  |
| Creatininaemia relative changes (Before surgery/M3)^f^, estimation [95% CI] |  |  |
| Tumour size ≤4 cm | -1.76 [-6.24;2.72] | *0.3829* |
| Tumour size ]4–7] cm | 2.03 [-2.77;6.83] |  |
| Tumour size >7 cm | -3.44 [-11.48;4.58] |  |
| Non-imperative NSS indication | 0.29 [-3.33;3.91] | *0.6466* |
| Imperative NSS indication | -2.30 [-7.84;3.23] |  |
| Creatininaemia relative changes (Before surgery/M12)^f^, estimation [95% CI] |  |  |
| Tumour size ≤4 cm | -0.92 [-3.97;2.13] | *0.4470* |
| Tumour size ]4–7] cm | 1.95 [-1.53;5.42] |  |
| Tumour size >7 cm | -1.06 [-8.33;6.22] |  |
| Non-imperative NSS indication | 0.13 [-2.28;2.53] | *0.9941* |
| Imperative NSS indication | 0.39 [-4.09;4.88] |  |
| Post-operative acute renal failure^c^, OR [95% CI] |  |  |
| Tumour size ≤4 cm | 0.10 [0.03-0.33] | *0.2889* |
| Tumour size ]4–7] cm | 0.49 [0.14-1.72] |  |
| Tumour size >7 cm | 0.76 [0.09-6.56] |  |
| Non-imperative NSS indication | 0.12 [0.03-0.55] | *0.6913* |
| Imperative NSS indication | 0.26 [0.1-0.68] |  |
| New-onset CKD (D1-D3)^c,g^, OR [95% CI] |  |  |
| Tumour size ≤4 cm | 1.18 [0.6-2.34] | *0.6066* |
| Tumour size ]4–7] cm | 1.09 [0.53-2.25] |  |
| Tumour size >7 cm | 0.61 [0.16-2.27] |  |
| Non-imperative NSS indication | 1.36 [0.78-2.37] | *0.1640* |
| Imperative NSS indication | 0.5 [0.21-1.21] |  |
| New-onset CKD (M3)^c,g^, OR [95% CI] |  |  |
| Tumour size ≤4 cm | 0.92 [0.12-7.24] | *0.1145* |
| Tumour size ]4–7] cm | 0.29 [0.07-1.25] |  |
| Tumour size >7 cm | 0.01 [0-0.41] |  |
| Non-imperative NSS indication | 0.33 [0.11-1.03] | *0.6040* |
| Imperative NSS indication | 0.09 [0.01-0.87] |  |
| New-onset CKD (M12)^c,g^, OR [95% CI] |  |  |
| Tumour size ≤4 cm | 0.23 [0.07-0.82] | *0.6083* |
| Tumour size ]4–7] cm | 0.44 [0.12-1.60] |  |
| Tumour size >7 cm | 0.92 [0.06-14.06] |  |
| Non-imperative NSS indication | 0.46 [0.17-1.2] | *0.3903* |
| Imperative NSS indication | 0.10 [0.01-0.79] |  |
| Trifecta^c,h^, OR [95% CI] |  |  |
| Tumour size ≤4 cm | 1.2 [0.76-1.92] | *0.0102* |
| Tumour size ]4–7] cm | 0.65 [0.41-1.03] |  |
| Tumour size >7 cm | 0.29 [0.11-0.72] |  |
| Non-imperative NSS indication | 0.89 [0.62-1.27] | *0.2145* |
| Imperative NSS indication | 0.46 [0.24-0.9] |  |
| **Length of hospital stay**^i^, estimation [95% CI] |  |  |
| Tumour size ≤4 cm | 0.66 [0.61-0.71] | *0.0950* |
| Tumour size ]4–7] cm | 0.67 [0.62-0.74] |  |
| Tumour size >7 cm | 0.71 [0.6-0.84] |  |
| Non-imperative NSS indication | 0.67 [0.63-0.72] | *0.8775* |
| Imperative NSS indication | 0.65 [0.59-0.72] |  |

**Abbreviations:** CI, confidence interval; CKD, chronic kidney disease; D, day; GFR, glomerular filtration rate; HR, hazard ratio; M, month; NSS, nephron sparing surgery; OR, odds ratio.

^a^Adjusted for the following prognostic factors: tumour size, imperative NSS indication, age at surgery, ASA score, and BMI at diagnosis. Stratified analyses, estimations, HRs, and ORs were adjusted for the following prognostic factors: imperative NSS indication, age at surgery, ASA score, and BMI at diagnosis for data stratified by tumour size, and tumour size, age at surgery, ASA score, and BMI at diagnosis for data stratified by NSS indication.

^b^Surgical complications only. When the Clavien-Dindo grade^[[1]](#footnote-1)^ was not provided, data were considered to be missing.

^c^Since the mixed effects regression model failed to converge, the Firth’s penalization method was used.

^d^Occurrence of at least one complication within 30 days post-surgery.

^e^As the survival model failed to converge, the Firth’s penalization method was used.

^f^To comply with model hypotheses, relative changes were calculated after transformation of the measured values into natural logarithms.

^g^For patients with pre-operative stage I or II CKD only.

^h^Trifecta was defined as negative surgical margins + zero perioperative complications and a warm ischaemia time (WIT) ≤25 min.

^i^To comply with model hypotheses, data for the length of hospital stay were transformed into natural logarithms. Estimations were the exponential of the model parameter, interpreted as a multiplicative factor: <1 (>1) means a shorter (longer) length of stay with RPN in comparison to OPN.

^1^ Dindo D, Demartines N, Clavien PA. Classification of surgical complications: a new proposal with evaluation in a cohort of 6336 patients and results of a survey. Ann Surg. 2004;240(2):205-13. doi: 10.1097/01.sla.0000133083.54934.ae.)

1. [↑](#footnote-ref-1)
