# Supplementary material for: Comparison of open and robotic-assisted partial nephrectomy approaches using multicentric data (UroCCR-47 study)
Source: Sci Rep. 2022 Nov 8;12:18981. doi: 10.1038/s41598-022-22912-8 (PMC9643517; doi:10.1038/s41598-022-22912-8)
Supplement: Supplementary file 3 — Supplementary Information 3. [file 41598_2022_22912_MOESM3_ESM.docx]

Supplementary Table 2: Number of procedures at each center

| Center | N | OPN | RPN |
| --- | --- | --- | --- |
| Total | 1969 | 560 | 1409 |
| 1 | 130 | 130 | 0 |
| 2 | 2 | 2 | 0 |
| 3 | 14 | 11 | 3 |
| 4 | 78 | 42 | 36 |
| 5 | 240 | 150 | 90 |
| 6 | 124 | 21 | 103 |
| 7 | 77 | 1 | 76 |
| 8 | 379 | 45 | 334 |
| 9 | 4 | 3 | 1 |
| 10 | 17 | 2 | 15 |
| 11 | 96 | 21 | 75 |
| 12 | 164 | 62 | 102 |
| 13 | 71 | 0 | 71 |
| 14 | 6 | 0 | 6 |
| 15 | 261 | 42 | 219 |
| 16 | 56 | 24 | 32 |
| 17 | 12 | 12 | 0 |
| 18 | 69 | 51 | 18 |
| 19 | 170 | 16 | 154 |
| 20 | 101 | 27 | 74 |

Abbreviations: OPN, open partial nephrectomy; RPN, robotic-assisted partial nephrectomy
